# Buildings Contemplated

**Published:** 1914-09-12

**Authors:** 


### Buildings Contemplated.

BARR.—It has been decided by the Joint Committee in charge of the provision of a sanatorium for the counties of Stirling and Clackmannan that the site of the hospital shall be at Barr, near Stirling, and they are proceeding to the purchase of thirty-six acres of land at the price of £1,040.

BERWICK-ON-TWEED.—The Local Government Board have intimated to the Guardians that they cannot consent to the proposed expenditure of £4,000 on alterations to the workhouse. The Guardians are urged to build a new infirmary in the grounds of the workhouse.

BILLERICAY.—The Guardians have instructed the architect to prepare plans for additions to the infirmary at Billericay.

BLEAN.—The Blean Board of Guardians invite tenders for new drainage, additions, and alterations to the workhouse at Herne Common, near Herne Bay, Kent. Quantities may be obtained of the architect, Mr. A. A. Kemp, 3 Tower Parade, Whitstable, Kent.

EVESHAM.—At a meeting of the Evesham Joint Hospital Board plans and estimates for the enlargement of the administrative block were considered, and it was agreed to apply to the Local Government Board for a grant in aid of the cost and for sanction to a loan.

HULL.—The Local Government Board will hold an inquiry into an application of the Hull Town Council for sanction to a loan of £14,438 for the erection of a sanatorium and hospital on the Castle Estate, Cottingham.

IPSWICH.—An inquiry has been held by the Local Government Board into an application of the Corporation for sanction to borrow £8,000 for the extension of the isolation hospital.

